# Biomarkers as Proxies for Cognitive Reserve: the role of high density lipoprotein cholesterol in first episode of psychosis

**DOI:** 10.1192/j.eurpsy.2023.1331

**Published:** 2023-07-19

**Authors:** R. Magdaleno Herrero, N. Murillo-García, Á. Yorca-Ruiz, K. Neergaard, B. Crespo-Facorro, R. Ayesa-Arriola

**Affiliations:** 1 Mental Heatlh, Instituto de Investigación Marqués de Valdecilla (IDIVAL), Santander; 2 CIBERSAM, Madrid; 3 Psychiatry, Hospital Universitario Virgen del Rocío, Sevilla, Spain

## Abstract

**Introduction:**

The proxies used to compose cognitive reserve (CR) in first episode of psychosis (FEP) have varied in the literature. The development of FEP is linked to the peripheral pathways of the central nervous system (Leboyer *et al.* Psychopharmacology 2016; 233(9) 1651-60) Furthermore, schizophrenia has been linked to the metabolic system, indicating that alterations in the levels of biological parameters, in particular high-density lipoproteins (HDL) (Gjerde *et al.* Eur Arch Psychiatry Clin Neurosci 2020; 270(1) 49-58) cause worse global functioning and cognitive impairment (Adamowicz *et al.* J Clin Med 2020; 9(2) 537). Despite this knowledge, no research has considered the introduction of biomarkers as proxies for CR.

**Objectives:**

The present study aimed to create a quantifiable and objective CR index that adjusts for the multifactorial nature of FEP.

**Methods:**

We included 668 patients who had FEP and 217 healthy controls who were assessed for sociodemographic information and levels of biological parameters: waist circumference, hypertension and levels of HDL, triglycerides and glucose. The main analyses were multiple regression analysis, principal component analysis (PCA) and exploratory factor analysis (EFA).

**Results:**

Regression analyses showed that HDL was the top performing biological parameter in a model containing years of education and unemployment (F=11.80; p<0.001) while also outperforming other parameters in a correlation analysis with a composite of the same variables (r= 0.21; p<0.001). In EFA analyses combining all possible components, we found that the most optimal proxies for the composition of biological CR were years of education and HDL. The results using PCA indicated that biological CR would have a greater explanatory power for the phenomenon than classical CR, increasing 7.27% of the explanation for FEP patients and 16.08% for healthy controls.

**Conclusions:**

This article proposes an objective and quantifiable method to measure CR, taking into account endogenous and exogenous factors. This index, introducing biomarkers as proxies could provide a more accurate CR score for FEP patients.

**Disclosure of Interest:**

None Declared

